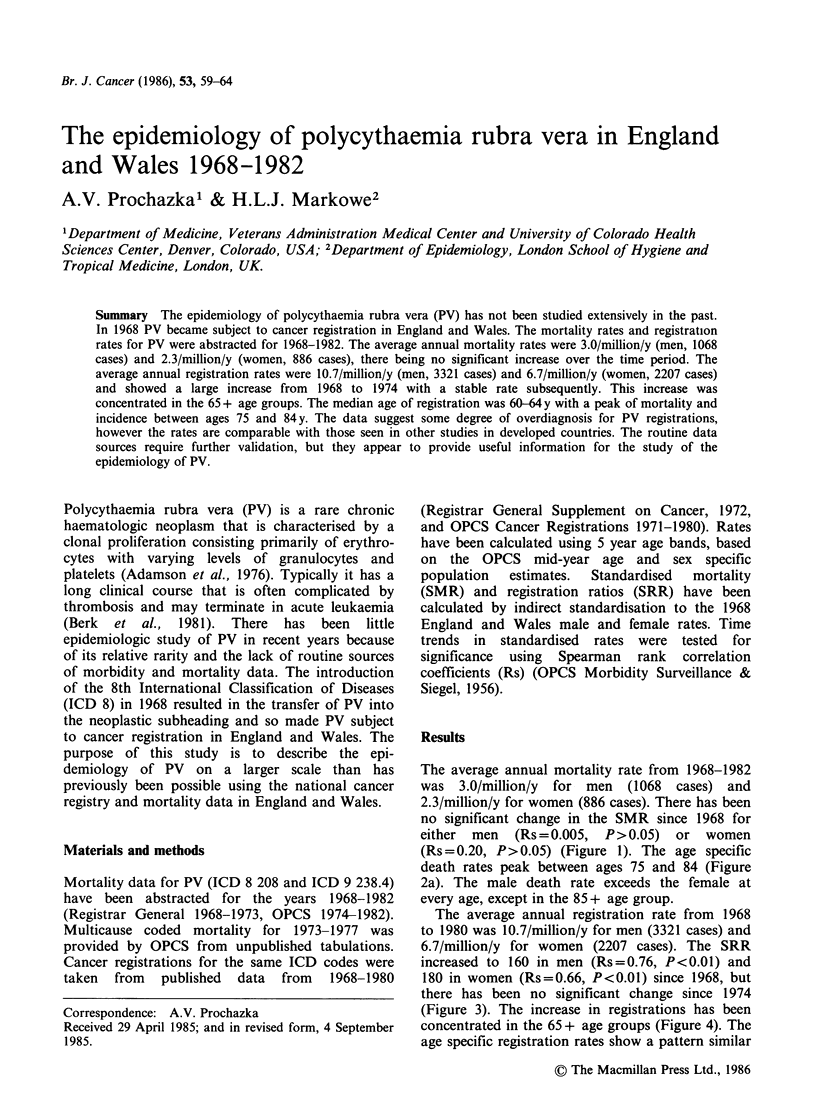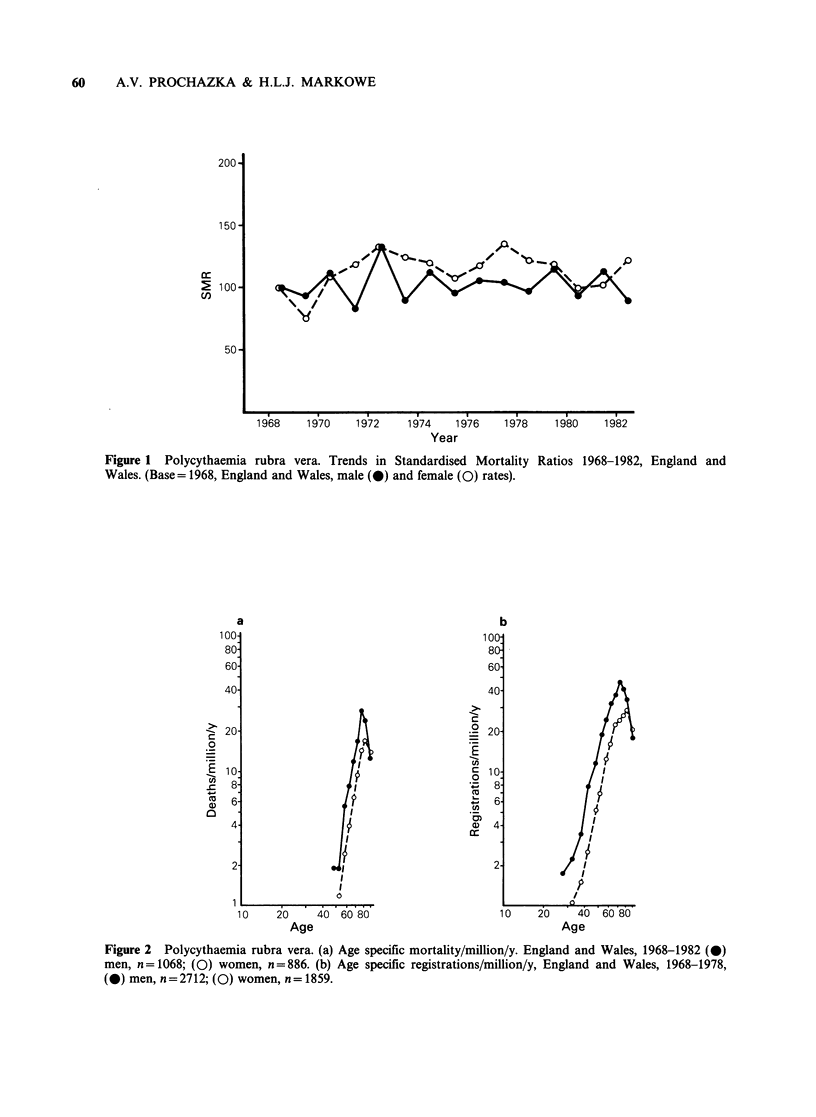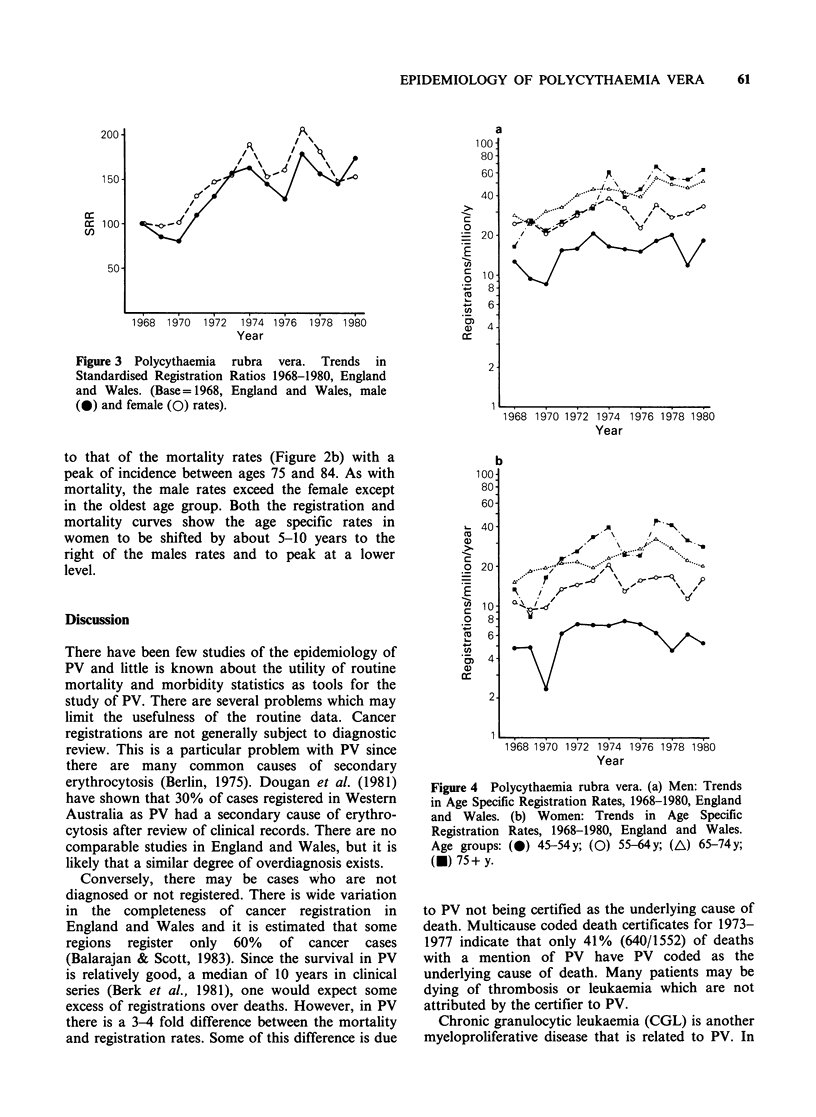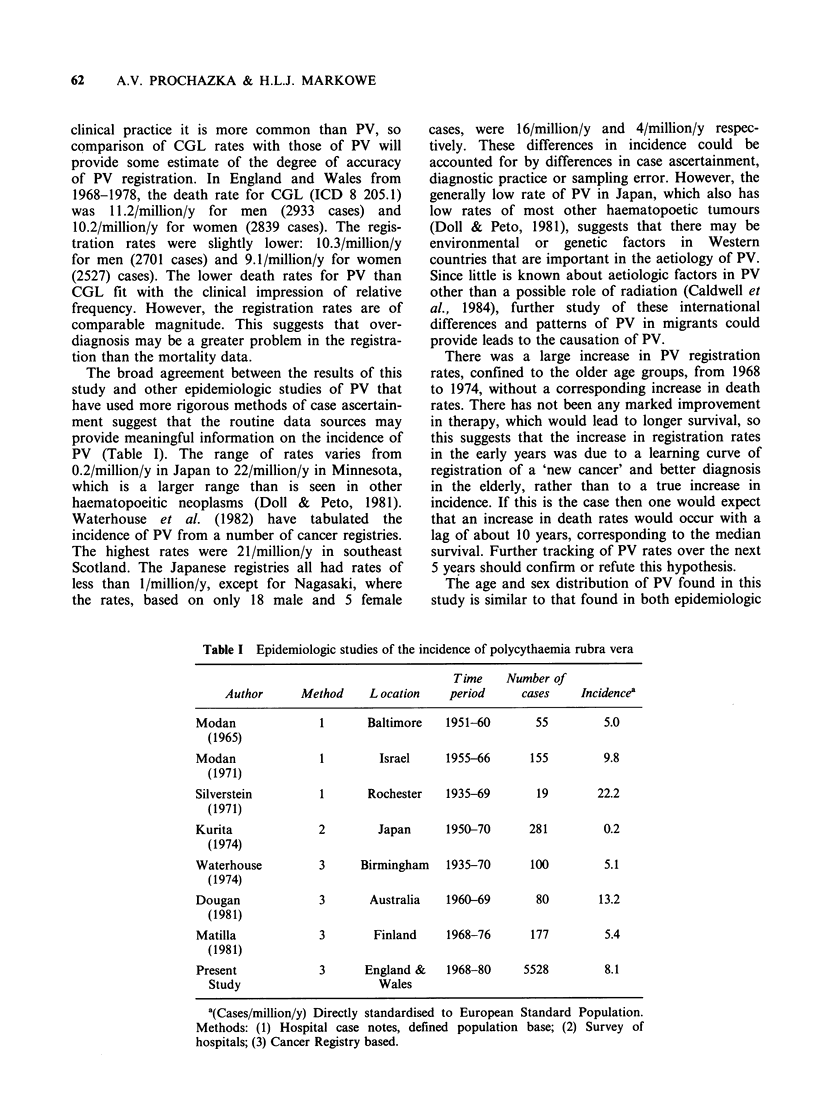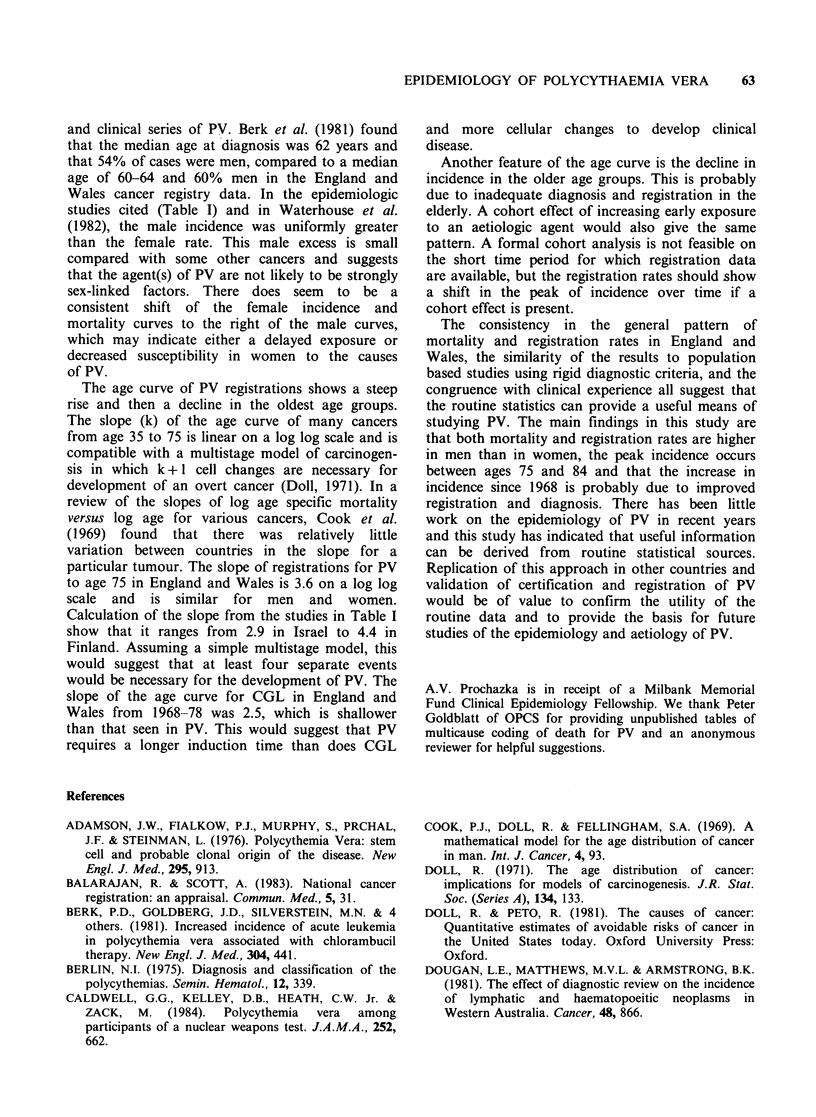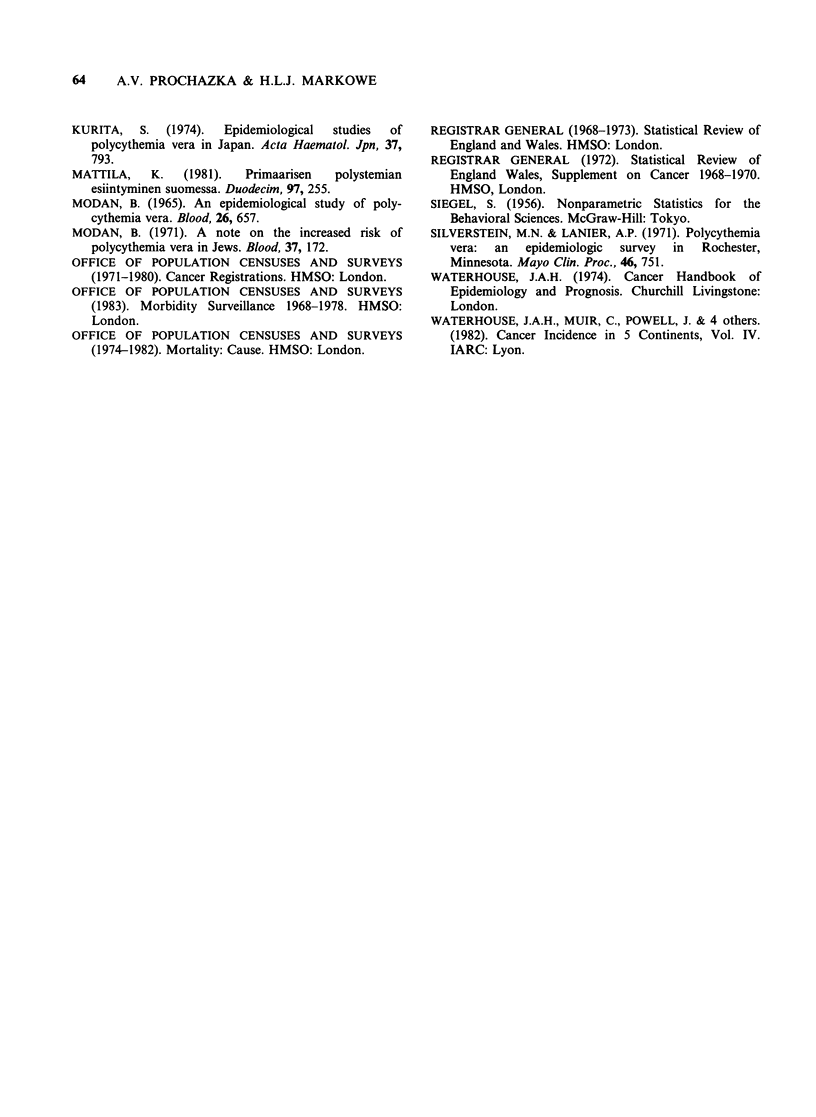# The epidemiology of polycythaemia rubra vera in England and Wales 1968-1982.

**DOI:** 10.1038/bjc.1986.9

**Published:** 1986-01

**Authors:** A. V. Prochazka, H. L. Markowe

## Abstract

The epidemiology of polycythaemia rubra vera (PV) has not been studied extensively in the past. In 1968 PV became subject to cancer registration in England and Wales. The mortality rates and registration rates for PV were abstracted for 1968-1982. The average annual mortality rates were 3.0/million/y (men, 1068 cases) and 2.3/million/y (women, 886 cases), there being no significant increase over the time period. The average annual registration rates were 10.7/million/y (men, 3321 cases) and 6.7/million/y (women, 2207 cases) and showed a large increase from 1968 to 1974 with a stable rate subsequently. This increase was concentrated in the 65+ age groups. The median age of registration was 60--64 y with a peak of mortality and incidence between ages 75 and 84 y. The data suggest some degree of overdiagnosis for PV registrations, however the rates are comparable with those seen in other studies in developed countries. The routine data sources require further validation, but they appear to provide useful information for the study of the epidemiology of PV.


Br. J. Cancer (1986), 53, 59–64

### The epidemiology of polycythaemia rubra vera in England and Wales 1968–1982

A.V. Prochazka[1] & H.L.J. Markowe[2]

[1]*Department of Medicine, Veterans Administration Medical Center and University of Colorado Health Sciences Center, Denver, Colorado, USA;* [2]*Department of Epidemiology, London School of Hygiene and Tropical Medicine, London, UK.*

**Summary** The epidemiology of polycythaemia rubra vera (PV) has not been studied extensively in the past. In 1968 PV became subject to cancer registration in England and Wales. The mortality rates and registration rates for PV were abstracted for 1968–1982. The average annual mortality rates were 3.0/million/y (men, 1068 cases) and 2.3/million/y (women, 886 cases), there being no significant increase over the time period. The average annual registration rates were 10.7/million/y (men, 3321 cases) and 6.7/million/y (women, 2207 cases) and showed a large increase from 1968 to 1974 with a stable rate subsequently. This increase was concentrated in the 65+ age groups. The median age of registration was 60–64 y with a peak of mortality and incidence between ages 75 and 84 y. The data suggest some degree of overdiagnosis for PV registrations, however the rates are comparable with those seen in other studies in developed countries. The routine data sources require further validation, but they appear to provide useful information for the study of the epidemiology of PV.

Polycythaemia rubra vera (PV) is a rare chronic haematologic neoplasm that is characterised by a clonal proliferation consisting primarily of erythrocytes with varying levels of granulocytes and platelets (Adamson *et al.*, 1976). Typically it has a long clinical course that is often complicated by thrombosis and may terminate in acute leukaemia (Berk *et al.*, 1981). There has been little epidemiologic study of PV in recent years because of its relative rarity and the lack of routine sources of morbidity and mortality data. The introduction of the 8th International Classification of Diseases (ICD 8) in 1968 resulted in the transfer of PV into the neoplastic subheading and so made PV subject to cancer registration in England and Wales. The purpose of this study is to describe the epidemiology of PV on a larger scale than has previously been possible using the national cancer registry and mortality data in England and Wales.

### Materials and methods

Mortality data for PV (ICD 8 208 and ICD 9 238.4) have been abstracted for the years 1968–1982 (Registrar General 1968–1973, OPCS 1974–1982). Multicause coded mortality for 1973–1977 was provided by OPCS from unpublished tabulations. Cancer registrations for the same ICD codes were taken from published data from 1968–1980 (Registrar General Supplement on Cancer, 1972, and OPCS Cancer Registrations 1971–1980). Rates have been calculated using 5 year age bands, based on the OPCS mid-year age and sex specific population estimates. Standardised mortality (SMR) and registration ratios (SRR) have been calculated by indirect standardisation to the 1968 England and Wales male and female rates. Time trends in standardised rates were tested for significance using Spearman rank correlation coefficients (Rs) (OPCS Morbidity Surveillance & Siegel, 1956).

Correspondence: A.V. Prochazka
Received 29 April 1985; and in revised form, 4 September 1985.

### Results

The average annual mortality rate from 1968–1982 was 3.0/million/y for men (1068 cases) and 2.3/million/y for women (886 cases). There has been no significant change in the SMR since 1968 for either men (Rs=0.005, $P>0.05$) or women (Rs=0.20, $P>0.05$) (Figure 1). The age specific death rates peak between ages 75 and 84 (Figure 2a). The male death rate exceeds the female at every age, except in the 85+ age group.

The average annual registration rate from 1968 to 1980 was 10.7/million/y for men (3321 cases) and 6.7/million/y for women (2207 cases). The SRR increased to 160 in men (Rs=0.76, $P<0.01$) and 180 in women (Rs=0.66, $P<0.01$) since 1968, but there has been no significant change since 1974 (Figure 3). The increase in registrations has been concentrated in the 65+ age groups (Figure 4). The age specific registration rates show a pattern similar

© The Macmillan Press Ltd., 1986

**Figure 1** Polycythaemia rubra vera. Trends in Standardised Mortality Ratios 1968–1982, England and Wales. (Base = 1968, England and Wales, male (●) and female (○) rates).

**Figure 2** Polycythaemia rubra vera. (a) Age specific mortality/million/y. England and Wales, 1968–1982 (●) men, $n = 1068$; (○) women, $n = 886$. (b) Age specific registrations/million/y, England and Wales, 1968–1978, (●) men, $n = 2712$; (○) women, $n = 1859$.

**Figure 3** Polycythaemia rubra vera. Trends in Standardised Registration Ratios 1968–1980, England and Wales. (Base = 1968, England and Wales, male (●) and female (○) rates).

**Figure 4** Polycythaemia rubra vera. (a) Men: Trends in Age Specific Registration Rates, 1968–1980, England and Wales. (b) Women: Trends in Age Specific Registration Rates, 1968–1980, England and Wales. Age groups: (●) 45–54 y; (○) 55–64 y; (△) 65–74 y; (■) 75+ y.

to that of the mortality rates (Figure 2b) with a peak of incidence between ages 75 and 84. As with mortality, the male rates exceed the female except in the oldest age group. Both the registration and mortality curves show the age specific rates in women to be shifted by about 5–10 years to the right of the males rates and to peak at a lower level.

### Discussion

There have been few studies of the epidemiology of PV and little is known about the utility of routine mortality and morbidity statistics as tools for the study of PV. There are several problems which may limit the usefulness of the routine data. Cancer registrations are not generally subject to diagnostic review. This is a particular problem with PV since there are many common causes of secondary erythrocytosis (Berlin, 1975). Dougan *et al.* (1981) have shown that 30% of cases registered in Western Australia as PV had a secondary cause of erythrocytosis after review of clinical records. There are no comparable studies in England and Wales, but it is likely that a similar degree of overdiagnosis exists.

Conversely, there may be cases who are not diagnosed or not registered. There is wide variation in the completeness of cancer registration in England and Wales and it is estimated that some regions register only 60% of cancer cases (Balarajan & Scott, 1983). Since the survival in PV is relatively good, a median of 10 years in clinical series (Berk *et al.*, 1981), one would expect some excess of registrations over deaths. However, in PV there is a 3–4 fold difference between the mortality and registration rates. Some of this difference is due to PV not being certified as the underlying cause of death. Multicause coded death certificates for 1973–1977 indicate that only 41% (640/1552) of deaths with a mention of PV have PV coded as the underlying cause of death. Many patients may be dying of thrombosis or leukaemia which are not attributed by the certifier to PV.

Chronic granulocytic leukaemia (CGL) is another myeloproliferative disease that is related to PV. In clinical practice it is more common than PV, so comparison of CGL rates with those of PV will provide some estimate of the degree of accuracy of PV registration. In England and Wales from 1968–1978, the death rate for CGL (ICD 8 205.1) was 11.2/million/y for men (2933 cases) and 10.2/million/y for women (2839 cases). The registration rates were slightly lower: 10.3/million/y for men (2701 cases) and 9.1/million/y for women (2527) cases). The lower death rates for PV than CGL fit with the clinical impression of relative frequency. However, the registration rates are of comparable magnitude. This suggests that over-diagnosis may be a greater problem in the registration than the mortality data.

The broad agreement between the results of this study and other epidemiologic studies of PV that have used more rigorous methods of case ascertainment suggest that the routine data sources may provide meaningful information on the incidence of PV (Table I). The range of rates varies from 0.2/million/y in Japan to 22/million/y in Minnesota, which is a larger range than is seen in other haematopoeitic neoplasms (Doll & Peto, 1981). Waterhouse *et al.* (1982) have tabulated the incidence of PV from a number of cancer registries. The highest rates were 21/million/y in southeast Scotland. The Japanese registries all had rates of less than 1/million/y, except for Nagasaki, where the rates, based on only 18 male and 5 female cases, were 16/million/y and 4/million/y respectively. These differences in incidence could be accounted for by differences in case ascertainment, diagnostic practice or sampling error. However, the generally low rate of PV in Japan, which also has low rates of most other haematopoetic tumours (Doll & Peto, 1981), suggests that there may be environmental or genetic factors in Western countries that are important in the aetiology of PV. Since little is known about aetiologic factors in PV other than a possible role of radiation (Caldwell *et al.*, 1984), further study of these international differences and patterns of PV in migrants could provide leads to the causation of PV.

There was a large increase in PV registration rates, confined to the older age groups, from 1968 to 1974, without a corresponding increase in death rates. There has not been any marked improvement in therapy, which would lead to longer survival, so this suggests that the increase in registration rates in the early years was due to a learning curve of registration of a 'new cancer' and better diagnosis in the elderly, rather than to a true increase in incidence. If this is the case then one would expect that an increase in death rates would occur with a lag of about 10 years, corresponding to the median survival. Further tracking of PV rates over the next 5 years should confirm or refute this hypothesis.

The age and sex distribution of PV found in this study is similar to that found in both epidemiologic

**Table I** Epidemiologic studies of the incidence of polycythaemia rubra vera

| *Author* | *Method* | *Location* | *Time period* | *Number of cases* | *Incidence*[a] |
|---|---|---|---|---|---|
| Modan (1965) | 1 | Baltimore | 1951–60 | 55 | 5.0 |
| Modan (1971) | 1 | Israel | 1955–66 | 155 | 9.8 |
| Silverstein (1971) | 1 | Rochester | 1935–69 | 19 | 22.2 |
| Kurita (1974) | 2 | Japan | 1950–70 | 281 | 0.2 |
| Waterhouse (1974) | 3 | Birmingham | 1935–70 | 100 | 5.1 |
| Dougan (1981) | 3 | Australia | 1960–69 | 80 | 13.2 |
| Matilla (1981) | 3 | Finland | 1968–76 | 177 | 5.4 |
| Present Study | 3 | England & Wales | 1968–80 | 5528 | 8.1 |

[a](Cases/million/y) Directly standardised to European Standard Population. Methods: (1) Hospital case notes, defined population base; (2) Survey of hospitals; (3) Cancer Registry based.

and clinical series of PV. Berk *et al.* (1981) found that the median age at diagnosis was 62 years and that 54% of cases were men, compared to a median age of 60–64 and 60% men in the England and Wales cancer registry data. In the epidemiologic studies cited (Table I) and in Waterhouse *et al.* (1982), the male incidence was uniformly greater than the female rate. This male excess is small compared with some other cancers and suggests that the agent(s) of PV are not likely to be strongly sex-linked factors. There does seem to be a consistent shift of the female incidence and mortality curves to the right of the male curves, which may indicate either a delayed exposure or decreased susceptibility in women to the causes of PV.

The age curve of PV registrations shows a steep rise and then a decline in the oldest age groups. The slope (k) of the age curve of many cancers from age 35 to 75 is linear on a log log scale and is compatible with a multistage model of carcinogensis in which $k+1$ cell changes are necessary for development of an overt cancer (Doll, 1971). In a review of the slopes of log age specific mortality *versus* log age for various cancers, Cook *et al.* (1969) found that there was relatively little variation between countries in the slope for a particular tumour. The slope of registrations for PV to age 75 in England and Wales is 3.6 on a log log scale and is similar for men and women. Calculation of the slope from the studies in Table I show that it ranges from 2.9 in Israel to 4.4 in Finland. Assuming a simple multistage model, this would suggest that at least four separate events would be necessary for the development of PV. The slope of the age curve for CGL in England and Wales from 1968–78 was 2.5, which is shallower than that seen in PV. This would suggest that PV requires a longer induction time than does CGL and more cellular changes to develop clinical disease.

Another feature of the age curve is the decline in incidence in the older age groups. This is probably due to inadequate diagnosis and registration in the elderly. A cohort effect of increasing early exposure to an aetiologic agent would also give the same pattern. A formal cohort analysis is not feasible on the short time period for which registration data are available, but the registration rates should show a shift in the peak of incidence over time if a cohort effect is present.

The consistency in the general pattern of mortality and registration rates in England and Wales, the similarity of the results to population based studies using rigid diagnostic criteria, and the congruence with clinical experience all suggest that the routine statistics can provide a useful means of studying PV. The main findings in this study are that both mortality and registration rates are higher in men than in women, the peak incidence occurs between ages 75 and 84 and that the increase in incidence since 1968 is probably due to improved registration and diagnosis. There has been little work on the epidemiology of PV in recent years and this study has indicated that useful information can be derived from routine statistical sources. Replication of this approach in other countries and validation of certification and registration of PV would be of value to confirm the utility of the routine data and to provide the basis for future studies of the epidemiology and aetiology of PV.

A.V. Prochazka is in receipt of a Milbank Memorial Fund Clinical Epidemiology Fellowship. We thank Peter Goldblatt of OPCS for providing unpublished tables of multicause coding of death for PV and an anonymous reviewer for helpful suggestions.

### References

ADAMSON, J.W., FIALKOW, P.J., MURPHY, S., PRCHAL, J.F. & STEINMAN, L. (1976). Polycythemia Vera: stem cell and probable clonal origin of the disease. *New Engl. J. Med.*, **295**, 913.

BALARAJAN, R. & SCOTT, A. (1983). National cancer registration: an appraisal. *Commun. Med.*, **5**, 31.

BERK, P.D., GOLDBERG, J.D., SILVERSTEIN, M.N. & 4 others. (1981). Increased incidence of acute leukemia in polycythemia vera associated with chlorambucil therapy. *New Engl. J. Med.*, **304**, 441.

BERLIN, N.I. (1975). Diagnosis and classification of the polycythemias. *Semin. Hematol.*, **12**, 339.

CALDWELL, G.G., KELLEY, D.B., HEATH, C.W. Jr. & ZACK, M. (1984). Polycythemia vera among participants of a nuclear weapons test. *J.A.M.A.*, **252**, 662.

COOK, P.J., DOLL, R. & FELLINGHAM, S.A. (1969). A mathematical model for the age distribution of cancer in man. *Int. J. Cancer*, **4**, 93.

DOLL, R. (1971). The age distribution of cancer: implications for models of carcinogenesis. *J.R. Stat. Soc. (Series A)*, **134**, 133.

DOLL, R. & PETO, R. (1981). The causes of cancer: Quantitative estimates of avoidable risks of cancer in the United States today. Oxford University Press: Oxford.

DOUGAN, L.E., MATTHEWS, M.V.L. & ARMSTRONG, B.K. (1981). The effect of diagnostic review on the incidence of lymphatic and haematopoeitic neoplasms in Western Australia. *Cancer*, **48**, 866.

KURITA, S. (1974). Epidemiological studies of polycythemia vera in Japan. *Acta Haematol. Jpn*, **37**, 793.

MATTILA, K. (1981). Primaarisen polystemian esiintyminen suomessa. *Duodecim*, **97**, 255.

MODAN, B. (1965). An epidemiological study of polycythemia vera. *Blood*, **26**, 657.

MODAN, B. (1971). A note on the increased risk of polycythemia vera in Jews. *Blood*, **37**, 172.

OFFICE OF POPULATION CENSUSES AND SURVEYS (1971-1980). Cancer Registrations. HMSO: London.

OFFICE OF POPULATION CENSUSES AND SURVEYS (1983). Morbidity Surveillance 1968–1978. HMSO: London.

OFFICE OF POPULATION CENSUSES AND SURVEYS (1974–1982). Mortality: Cause. HMSO: London.

REGISTRAR GENERAL (1968–1973). Statistical Review of England and Wales. HMSO: London.

REGISTRAR GENERAL (1972). Statistical Review of England Wales, Supplement on Cancer 1968–1970. HMSO, London.

SIEGEL, S. (1956). Nonparametric Statistics for the Behavioral Sciences. McGraw-Hill: Tokyo.

SILVERSTEIN, M.N. & LANIER, A.P. (1971). Polycythemia vera: an epidemiologic survey in Rochester, Minnesota. *Mayo Clin. Proc.*, **46**, 751.

WATERHOUSE, J.A.H. (1974). Cancer Handbook of Epidemiology and Prognosis. Churchill Livingstone: London.

WATERHOUSE, J.A.H., MUIR, C., POWELL, J. & 4 others. (1982). Cancer Incidence in 5 Continents, Vol. IV. IARC: Lyon.